# Elucidation of the Mechanism of Rapid Growth Recovery in Rice Seedlings after Exposure to Low-Temperature Low-Light Stress: Analysis of Rice Root Transcriptome, Metabolome, and Physiology

**DOI:** 10.3390/ijms242417359

**Published:** 2023-12-11

**Authors:** Xiaoquan Fu, Lei Zhong, Hui Wang, Haohua He, Xiaorong Chen

**Affiliations:** Key Laboratory of Crop Physiology, Ecology and Genetic Breeding, Ministry of Education, Jiangxi Agricultural University, Nanchang 330045, China; fxq11111720@163.com (X.F.); zhonglei2000@163.com (L.Z.); huiwang1997@126.com (H.W.); hhhua64@163.com (H.H.)

**Keywords:** rice, late spring cold, growth recovery, root system, IAA, sucrose

## Abstract

Late spring cold is a disastrous weather condition that often affects early rice seedlings in southern China, limiting the promotion of direct seeding cultivation. However, there are few reports on the effect of these events and on the growth recovery mechanism of rice root systems after rice seedlings are exposed to this stress. This study selected the strong-growth-recovery variety B116 (R310/R974, F_17_) and the slow-recovery variety B811 (Zhonghui 286) for direct seeding cultivation and exposed them to low temperature and low-light stress to simulate a late spring cold event in an artificial climate chamber. The treatment consisted of 4 days of exposure to a day/night temperature of 14/10 °C and a light intensity of 266 µmol m^−2^s^−1^ while the control group was kept at a day/night temperature of 27/25 °C and light intensity of 533 µmol m^−2^s^−1^. The results showed that 6 days after stress, the total length, surface area, and volume of B116 roots increased by 335.5%, 290.1%, and 298.5%, respectively, while those of B811 increased by 228.8%, 262.0%, and 289.1%, respectively. In B116, the increase in root fresh weight was 223.1%, and that in B811 was 165.6%, demonstrating rapid root recovery after stress and significant differences among genotypes. The content of H_2_O_2_ and MDA in the B116 roots decreased faster than that in the B811 roots after normal light intensity and temperature conditions were restored, and the activity of ROS metabolism enzymes was stronger in B116 roots than in B811 roots. The correlation analysis between the transcriptome and metabolome showed that endogenous signal transduction and starch and sucrose metabolism were the main metabolic pathways affecting the rapid growth of rice seedling roots after exposure to combined stress from low temperature and low light intensities. The levels of auxin and sucrose in the roots of the strong-recovery variety B116 were higher, and this variety’s metabolism was downregulated significantly faster than that of B811. The auxin response factor and sucrose synthesis-related genes *SPS1* and *SUS4* were significantly upregulated. This study contributes to an understanding of the rapid growth recovery mechanism in rice after exposure to combined stress from low-temperature and low-light conditions.

## 1. Introduction

Rice is a major food crop worldwide. Direct seeding has become an important method for early rice production in southern China due to its simplicity, speed, and cost-effectiveness [1,2]. The sowing time for early rice is generally from mid- to late March, which coincides with the occurrence of “late spring cold”, a weather phenomenon involving sudden cooling and warming accompanied by a decrease in light intensity. This phenomenon results in an average daily temperature of 10–14 °C [3], causing the rapid cooling and warming of rice seedlings. Studies have shown that low temperatures at the seedling stage negatively impact rice yield and increase weed damage in fields [4,5,6]. Additionally, due to global warming, the sowing date for early rice has advanced, leading to an increased risk of cold damage for directly seeded early rice.

Low temperature events often cause nonlethal damage, resulting in slow growth recovery in seedlings, reduced yield, and an extended growth period [7,8]. Weak and slow growth in rice seedlings has become a leading cause of yield reduction in cold-water rice areas [9]. However, plants tend to grow rapidly after mild stress; this is known as compensatory growth in ecology. The effect of single low-temperature stress events on rice yield and the related mechanisms are increasingly clear [10,11], but the growth recovery mechanism of directly seeded early rice after combined stress from low temperature and low light is rarely reported.

The growth status of rice root systems is crucial for rice seedlings [12]. Research shows that the absorption capacity of roots is significantly correlated with root length and surface area [13]. Longer and deeper root-system growth is induced in the soil by appropriate drought to obtain greater root absorption areas [14]. After low-temperature stress, the root system of rice seedlings experiences significant reductions in indicators such maximum root length, total root surface area, and total root volume [15,16], inhibiting material accumulation and subsequent growth and development [17]. However, research on the root growth recovery of rice seedlings after combined stress from low temperature and low light intensity in late spring is limited, and there are no reports on the differences and mechanisms of root growth recovery after stress among varieties.

Reactive oxygen species (ROS) production is often the main cause of plant growth reduction [18]. ROS can disrupt material metabolism and cause adverse physiological and biochemical reactions [19]. The ROS scavenging system can remove ROS in a timely manner after a low-temperature event followed by a high-temperature stress event, ensuring growth recovery in plants [18,20]. After low-temperature stress, the accumulation of reactive oxygen species in rice seedling roots increases, affecting the growth of rice seedlings and gradually stabilizing in the recovery process [21].

Recent studies have employed combined multi-omics analyses to elucidate biological phenomena [22,23,24]. Through transcriptome association analysis, it was found that genes related to metabolic pathways in rice underwent significant changes after low-temperature stress [25]. Extensive metabolomic studies showed that sugars were significantly enriched, and their content increased after low-temperature stress in rice [26].

The changes in root length and surface area after stress are related to whether the plant can grow and recover rapidly. A rapid recovery process occurs after combined stress from low temperature and low light intensity in the aboveground parts of rice [25]. Therefore, it is believed that a rapid recovery process also occurs after combined stress from low temperature and low light intensity in the seedling stage of rice root growth. Hormone signal transduction, ROS metabolism, and nitrogen absorption are speculated to play a major role in the recovery of rice root growth. The elimination of ROS is key to the recovery of root growth after low-temperature stress. Additionally, root cell division is predicted to intensify to expand the available surface area for root absorption. The specific parameters affected will be root length and the number of main roots.

To clarify the rapid growth recovery mechanisms of rice roots after exposure to combined stress from low temperature and low light at the seedling stage, a study was conducted using direct rice seeding and an artificial climate chamber to simulate the low-temperature low-light conditions associated with “late spring cold”. The study focused on rice root physiology, gene expression, and metabolites, as well as on the changes that occur in root growth during growth recovery after a stress event, with special emphasis on the differences among varieties. The purpose of this study was to provide a theoretical basis for the breeding and cultivation of direct seeded rice exposed to late spring cold.

## 2. Results

### 2.1. Root Growth Status of Rice Seedlings during Growth Recovery after Combined Stress from Low Temperatures and Low Light Intensities

**Figure 1:**
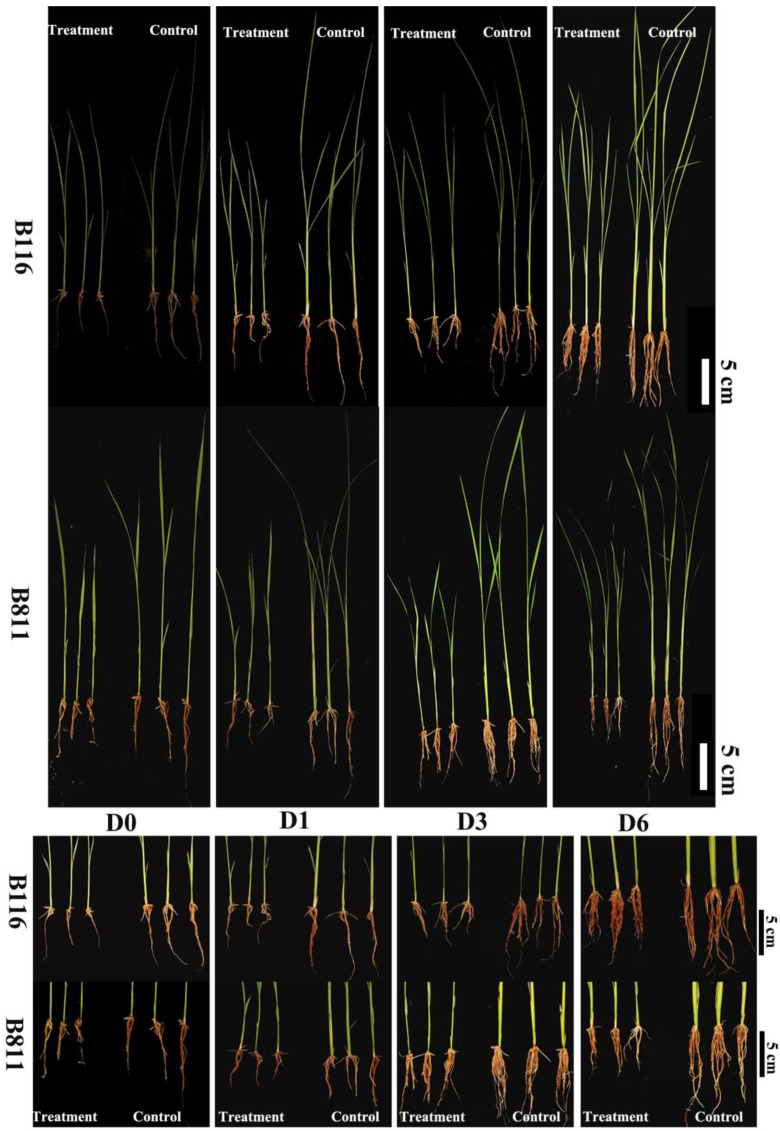
Root growth in rice seedlings is at different recovery stages. D0, D1, D3, and D6 represent Days 0, 1, 3, and 6, respectively, after the light and temperature conditions were restored to a normal state. B116 and B811 represent two different varieties. In the image, “Treatment” indicates the group experiencing combined stress from low temperature and low light intensity, and” Control” indicates the normal light and temperature group.

**Figure 2:**
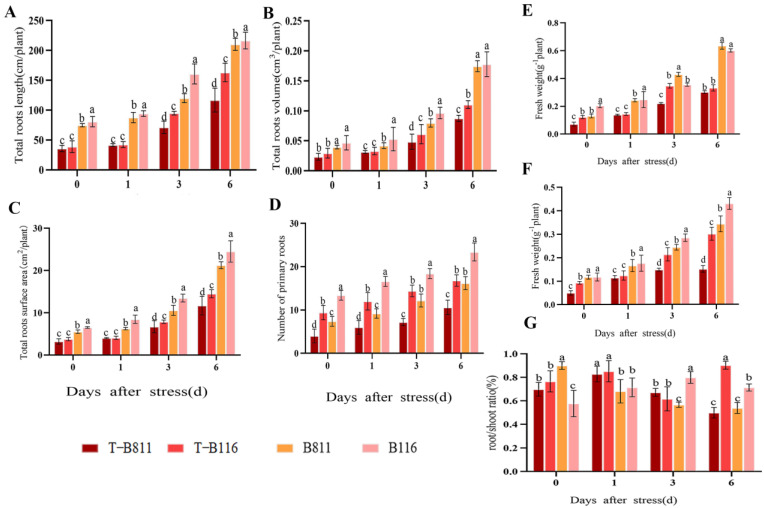
Root scan results and fresh weight. (**A**) Total root length (cm): we removed all the roots of a rice seedling intact and placed them on a flat surface. The scanner performed scanning imaging, and we analyzed the images by adding up the lengths of all the roots to arrive at the total length of the root system of a seedling. (**B**) Total root volume (cm^3^) denotes the total volume of the root system of a single rice seedling as derived from the scan. (**C**) Total root surface area (cm^2^) denotes the total surface area of the root system of a single rice seedling as derived from the scan. (**D**) Total root number, indicating the number of primary roots in the root system of a single rice seedling. (**E**) Fresh weight of aboveground parts per plant (g). (**F**) Fresh root weight per plant (g). (**G**) Fresh weight root to shoot ratio. “T” indicates treatment, i.e., low-temperature and low-light conditions. No “T” indicates control, i.e., normal light and temperature conditions. B116 and B811 indicate the two tested varieties. Error bars represent standard deviation (*n* = 3). Data are means ± SD. Different lowercase letters indicate significant differences at *p* < 0.05 according to Duncan’s test.

After being exposed to combined stress from low temperatures and low light intensities, the roots of two rice varieties showed varying degrees of lag that were significantly different from the control (Figure 1). The total root lengths of the two varieties were 51.7% and 52.1% less than that of the control on the 0th day after normal light and temperature conditions were restored. B116 roots were only 25.3% shorter than the control roots on the 6th day after normal light and temperature conditions were restored, while B811 roots were 44.3% shorter (Figure 2A). After B116 returned to normal light and temperature conditions, the difference between its number of main roots and that of the control did not decrease (Figure 2D) and remained at approximately 28.0%. In contrast, that value in B811 decreased from 45.9% on the 0th day to 34.5%, indicating that the total root length of B116 recovered rapidly after the restoration of normal light and temperature conditions, while in B811, mainly, the number of main roots increased. The total root volume and total root surface area showed the same trend as the total root length (Figure 2B,C). After normal light and temperature conditions were restored following stress conditions, the fresh weight of B116 roots increased by 223.1% on Day 6 compared with the 0th day (Figure 2F), while that of B811 increased by 165.6%, and the difference was significant. It is worth noting that although the fresh weight recovered, the gap between the treatment and the control increased. The difference in fresh weight between B116 and T-B116 increased from 10.6% on the 0th day to 31.6%, while that of B811 increased to 55.1%. This might have occurred because the number and length of roots increased first after exposure to low-temperature low-light stress, but the root quality did not rapidly improve, and root thickness was not high.

### 2.2. Reactive Oxygen Species Metabolism in Rice Seedling Roots during Growth Recovery after Combined Stress from Low Temperatures and Low Light Intensities

**Figure 3:**
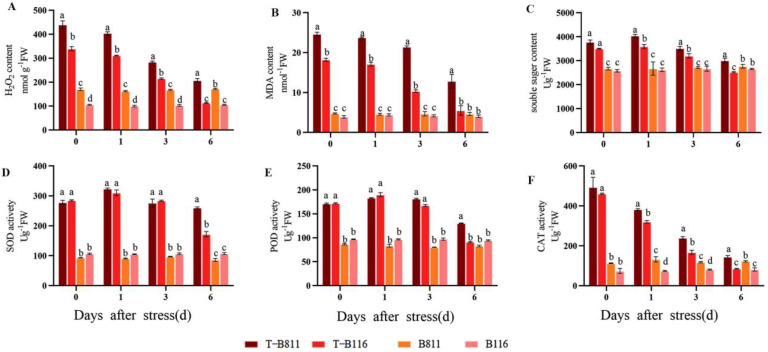
Reactive oxygen species accumulation and metabolic enzyme activities in rice seedling roots at different recovery stages. (**A**) Hydrogen peroxide (H_2_O_2_) content (nmol g^−1^FW); (**B**) Malondialdehyde (MDA) content (nmol^−1^FW); (**C**) soluble sugar content (Ug^−1^FW); (**D**) Superoxide dismutase (SOD) activity (Ug^−1^FW); (**E**) Peroxidase (POD) activity (Ug^−1^FW); (**F**) Catalase (CAT) activity (Ug^−1^FW). “T” indicates treatment, i.e., low-temperature and low-light conditions. No “T” indicates control, i.e., normal light and temperature conditions. B116 and B811 indicate the two tested varieties. Error bars represent standard deviation (*n* = 3). Data are means ± SD. Different lowercase letters indicate significant differences at *p* < 0.05 according to Duncan’s test.

The reactive-oxygen-species-related metabolites and metabolic enzyme activities of roots changed significantly after normal growth conditions were restored following combined stress from low temperatures and low light intensities (Figure 3). The H_2_O_2_ content of B116 roots was 221.1% higher than that of the control, while that of B811 roots was 158.2% higher (Figure 3). However, by the 6th day after normal light and temperature conditions were restored, the MDA content in the roots of B811 was still 21.2% higher than that of the control, while that of B116 was only 7.5% higher, showing a consistent change (Figure 3B). After six days of recovery, the MDA content in the roots of B116 was only 35.6% higher than that of the control, but that of B811 was still 176.6% higher. Soluble sugar increased first and then decreased during recovery under normal light and temperature conditions (Figure 3C). The H_2_O_2_ content in B116 recovered from being 35.7% higher than the control on the 0th day of growth recovery to 5.1%, while that in B811 increased from 41.6% to 51.2% and then recovered to 8.7% on the 6th day. After the restoration of normal temperature and light conditions, the activities of related metabolic enzymes in roots increased significantly compared with those in the control. They increased significantly over the first two days of the recovery period and began to decline on the third day. The POD activity of B116 increased from being 77.6% to 96.4% higher than that of the control on the 0th day and only 3.6% lower than that of the control on the 6th day. Meanwhile, that in B811 increased from 97.8% to 126.0%, and it dropped to 56.9% on the 6th day (Figure 3E). SOD activity and POD activity showed a consistent trend. The SOD activity of B116 was 60.5% higher than that of the control on the 6th day of recovery (Figure 3D) while the difference in the SOD activity of B811 between the 0th day and 6th day was not significantly lower, and it was approximately 201.7% higher than that of the control. The results showed that the increase in POD activity was the main manifestation of the ROS scavenging process in B811. CAT activity showed a downward trend (Figure 3F). Compared with the control, the CAT activity values of B116 and B811 were 4.3% and 17.9% higher than that of the control on the 6th day, respectively. However, immediately after normal light and temperature conditions were restored, the CAT activity of the B116 roots was 518.6% higher than that of the control while that of B811 was only 337.2% higher. Based on the changes in ROS and related metabolic enzyme activities, these results showed that the ROS scavenging ability of roots in the strong-growth-recovery variety B116 was stronger than that of the low-growth-recovery variety B811.

### 2.3. Differential Analysis of Gene Expression in Rice Seedling Roots during Growth Recovery after Combined Stress from Low Temperature and Low Light Intensity

**Figure 4:**
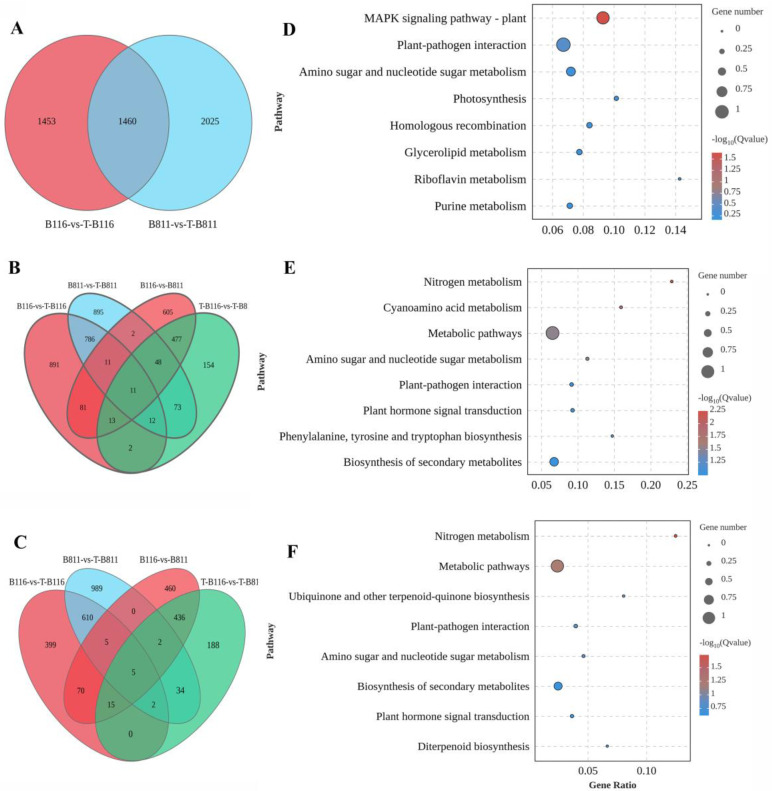
Analysis of differentially expressed genes and their functional enrichment. (**A**): Venn diagram of differentially expressed genes in B116−vs.−T−B116 and B811−vs.−T−B811 groups, (**B**): Venn diagram of upregulated genes, (**C**): Venn diagram of downregulated genes, (**D**): KEGG enrichment pathway for specific genes in B116−vs.−T−B116 group, (**E**): KEGG enrichment pathway for specific genes in B811−vs.−T−B811 group, (**F**): KEGG enrichment pathway for common genes in B116−vs.−T−B116 and B811−vs.−T−B811 groups.

After experiencing combined stress from low temperatures and low light intensities, significant differences were observed in root phenotype and ROS metabolism between B116 and B811. Both varieties reached a relatively stable state after 6 days of growth recovery. Therefore, gene expression was studied in B116 and B811, which have different growth recovery abilities, after 6 days of recovery under normal conditions. There were four comparison groups: B116-vs-T-B116, B811-vs-T-B811, B116-vs-B811, and T-B116-vs-T-B811. Prior to analysis, PCA was conducted on the samples, revealing significant differences between the samples and good clustering within the groups. The sequencing results showed that each sample produced an average of 612 million base pairs, with a Q20 ratio of 97.37%, a Q30 ratio of 93.03%, and an average GC content of 52.23%. These results indicated that the sequencing results were reliable and suitable for differential analysis (Table S1). Subsequently, differentially expressed genes (DEGs) were analyzed (FDR < 0.05, |log_2_ (FC)| > 1). In the B116-vs-T-B116 comparison group, 1807 differentially upregulated genes and 1106 differentially downregulated genes were enriched; the B811-vs-T-B811 group was enriched in 1838 differentially upregulated genes and 1647 differentially downregulated genes. The B116-vs-B811 group was enriched in 2241 differential genes (1248 upregulated and 993 downregulated), while the T-B116-vs-T-B811 group was enriched in 1472 differential genes (790 upregulated and 682 downregulated) (Figure 4A,B). After treatment, the number of differentially expressed genes across the two varieties was significantly lower than that when comparing the control and the treatment and control for a single variety, indicating that there were specific and co-expressed genes during the recovery from exposure to combined low-temperature and low-light stress. Therefore, further analysis was conducted of the B116-vs.-T-B116 and B811-vs.-T-B811 groups to analyze the differences between the unique and common gene sets of a single variety.

### 2.4. Functional Enrichment Analysis of Differentially Expressed Genes in Rice Seedling Roots during Growth Recovery after Combined Stress from Low Temperatures and Low Light Intensities

After the combined stress of low temperature and low light, normal conditions were restored, and a significant difference in differentially expressed genes (DEGs) between B116 and B811 was observed. There were 1460 co-differentially expressed genes in the B116−vs.−T−B116 and B811−vs.−T−B811 groups, with 1453 specific DEGs in the B116−vs.−T−B116 group and 2025 specific DEGs in the B811−vs.−T−B811 group (Figure 4C). In the phenotypic analysis, B116 was identified as a rapid-recovery type while B811 was a slow-recovery type. Therefore, the genes specifically expressed by the B116−vs.−T−B116 group were identified as the rapid-recovery gene set, and the genes specifically expressed by the B811−vs.−T−B811 group were identified as the slow-recovery gene set.

DEGs play a role in various metabolic pathways. To characterize the functions of the different genes in each set, KEGG functional enrichment analysis was performed on the different genes in each set. In the rapid growth recovery gene set, differentially expressed genes were mainly concentrated in “MAPK signal transduction pathway (plants)”, “plant pathogen interaction”, “photosynthesis”, and “amino sugar and nucleoside sugar metabolism” (Figure 4E). DEGs in the slow-recovery gene set were concentrated in metabolic pathways such as “nitrogen metabolism”, “metabolic pathways”, and “plant endogenous signal transduction” (Figure 4F). Function enrichment analysis of the common differentially expressed genes in the two comparison groups showed that the common differentially expressed genes were mainly enriched in “nitrogen metabolism”, “metabolic pathways”, “diterpenoid biosynthesis”, and “plant endogenous signal transduction” (Figure 4D). Therefore, there were obvious differences in metabolic pathways in different varieties during the growth recovery process after plant exposure to combined stress from low temperatures and low light intensities. The MAPK signal transduction pathway and photosynthesis may be related to rapid recovery, while nitrogen metabolism and plant endogenous signal transduction and diterpenoid biosynthesis may be related to slow recovery. In view of the obvious differences in metabolic pathways among varieties, we determined and analyzed plant metabolites.

### 2.5. Differential Analysis of Root Metabolites of Rice Seedlings during Growth Recovery after Exposure to Combined Stress from Low Temperatures and Low Light Intensities

**Figure 5:**
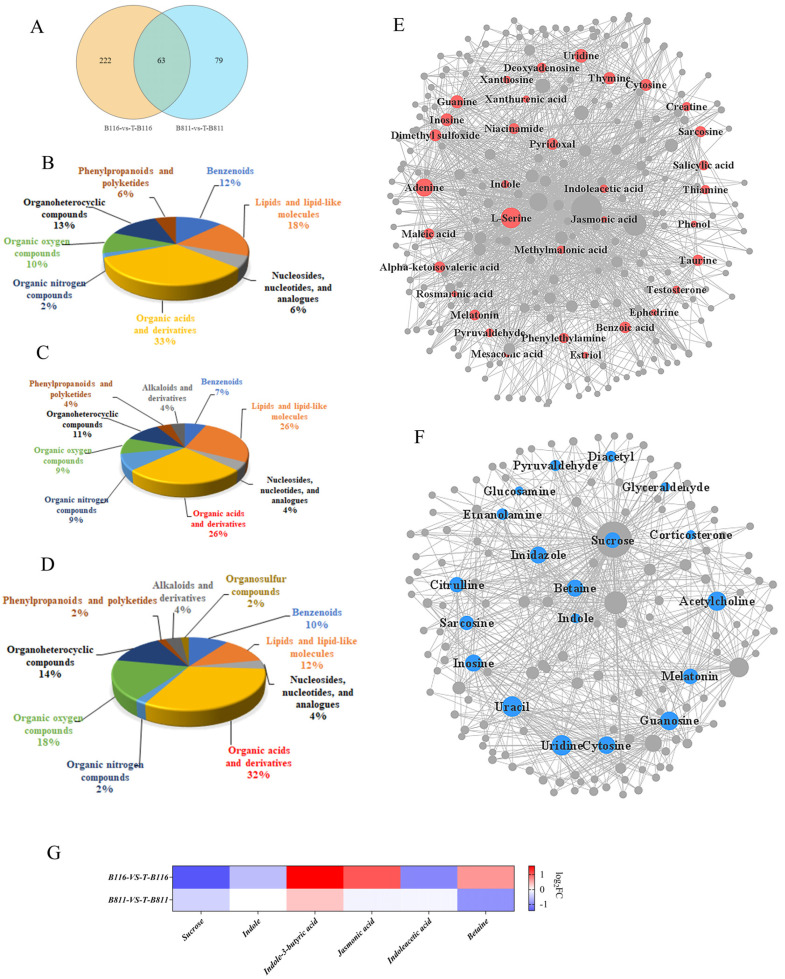
Analysis of differential metabolites. (**A**) Venn diagram of differentially abundant metabolites in B116−vs.-T−B116 and B811−vs. −T−B811 groups. (**B**) Classification of specific metabolite sets in B116−vs. −T−B116 group. (**C**) Classification of specific metabolite sets in B811−vs.−T−B811 group. (**D**) Classification of common differentially abundant metabolite sets in B116−vs.−T−B116 and B811−vs.−T−B811 groups. (**E**) Correlation analysis of differentially abundant metabolites in B116−vs.−T−B116 group. (**F**) Correlation analysis of differentially abundant metabolites in B811−vs.−T−B811 group. (**G**) Heatmap of key metabolite correlations based on material log_2_ FC.

Untargeted metabolomic analysis is a common method used to study stress and identify key metabolites. The transcriptomics results showed significant differences in metabolic pathways among B116, B811, T-B116 and T-B811 treatments. A total of 1351 known secondary metabolites were analyzed using UHPLC, including 864 positive ions and 487 negative ions. A total of 285 differentially abundant metabolites were detected in the B116-vs.-T-B116 comparison group (171 differentially upregulated and 114 differentially downregulated), and 142 differentially abundant metabolites were detected in the B811-vs.-T-B811 group (46 differentially upregulated and 96 differentially downregulated) (Figure 5A and Table S2). Based on the grouping scheme of the transcriptome, we divided the differentially abundant metabolites into three sets. In the rapid-growth-recovery set, we found that the main differentially abundant metabolites were organic acids and derivatives (33%) and lipids and lipid molecules (18%) (Figure 5B), while in the slow growth recovery set, also, they were organic acids and derivatives (32%) and lipids and lipid molecules (24%) (Figure 5C). In the common set, organic acids and derivatives were at 32% and organic oxygen compounds were at 18% (Figure 5D) (Table S2). The results showed that organic acids, lipids, and lipid molecules played an important role in the growth recovery of rice seedling roots after normal growth conditions were restored. The network diagram helps us elucidate the key metabolites that lead to different sources. Correlation analysis of the different metabolites in the B116-vs.-T-B116 and B811-vs.-T-B811 groups shows that serine, jasmonic acid, and indoleacetic acid play key roles in the recovery of root growth in B116 after low-temperature and low-light stress is relieved (Figure 5E and Table S3). In the B811-vs.-T-B811 comparison group, betaine and sucrose were identified as two key metabolites (Figure 5F and Table S4). By comparing the changes in several metabolites in the growth recovery of different treatments after low-temperature and low-light stress was relieved, it was found that the sucrose level of B116 cells was downregulated more than that of B811 cells, and auxin was significantly downregulated in B116 cells but not in B811 cells (Figure 5G).

### 2.6. Analysis of Key Genes and Metabolites for the Rapid Growth and Recovery of Rice Seedlings after Exposure to Combined Stress from Low Temperatures and Low Light Intensities

**Figure 6:**
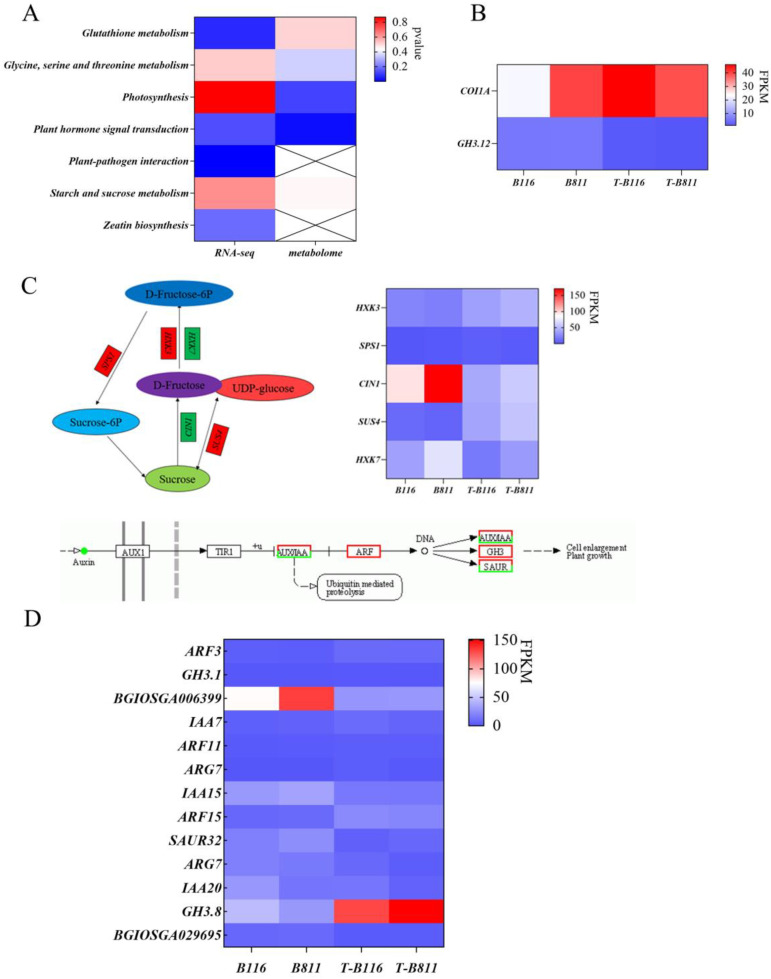
Association analysis of differential genes and differential metabolites. (**A**) Heatmap of *p* values for common pathways in the transcriptome and metabolism. (**B**) FPKM values for differential genes in the jasmonic acid signaling pathway. (**C**) FPKM values for the starch and sucrose metabolism pathway and its associated genes. Circles represent substances; rectangles represent genes, arrows indicate transitions between substances or pathways for signaling. (**D**) FPKM values for the auxin signaling pathway and its differentially expressed genes.

**Figure 7:**
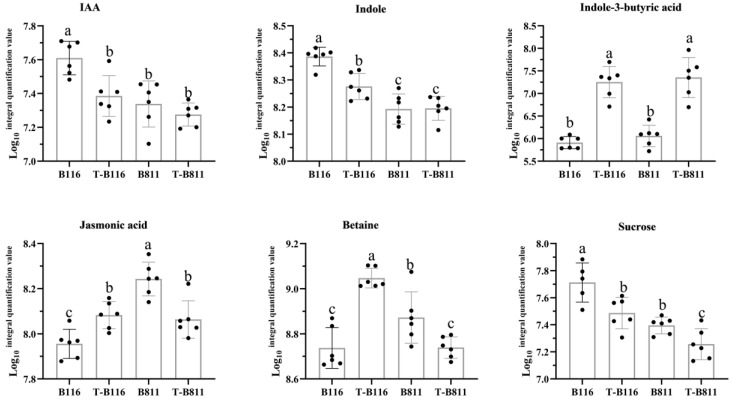
Differences in metabolic levels of key metabolites in different treatments. The abscissa represents each process, and the ordinate is the logarithm of the quantitative value of LC‒MS peak-area integration with the base of 10, the black dots indicate the specific values measured. “T” denotes treatment, i.e., low temperature and low light. No “T” denotes control, i.e., normal light and temperature. B116 and B811 denote the two tested varieties. Error bars represent standard deviation (*n* = 3). Data are means ± SD. Different lowercase letters indicate significant differences at *p* < 0.05 according to Duncan’s test.

**Figure 8:**
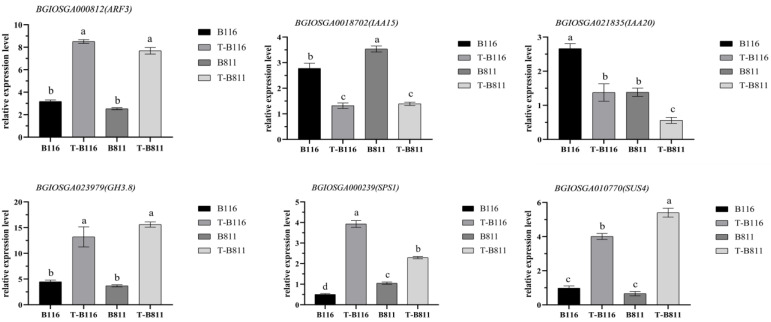
Real-time quantitative expression of RT-qPCR. “T” denotes treatment, i.e., low temperature and low light. No “T” denotes control, i.e., normal light and temperature. B116 and B811 denote the two test varieties. Error bars represent standard deviation (*n* = 3). Data are means ± SD. Different lowercase letters indicate significant differences at *p* < 0.05 according to Duncan’s test.

The analysis of differential expressed genes and metabolites helps us understand the key pathways and major metabolic species involved in rice seedling root growth recovery after relief from low-temperature and low-light stress. To identify the genes and metabolites that may play a major regulatory role in the process of root growth recovery, we conducted an association analysis of differential genes and metabolites. The results showed the significant enrichment of plant endogenous signal transduction and starch and sucrose metabolism in different genes and different metabolites (Figure 6A). Specifically, the auxin signaling pathway and jasmonic acid signaling pathway were identified as the main pathways for plant endogenous signal transduction. Additionally, the conversion between sucrose and fructose is another important pathway. Gene expression and metabolite-level analysis revealed the significant upregulation of the auxin response factors *ARF3*, *ARF11*, *ARF15*, and auxin amide synthase *GH3.8*, while the auxin response proteins *IAA20* and *IAA15* were significantly downregulated (Figure 6D). The jasmonic acid signal response factor *GH3.12* was significantly downregulated in both varieties (Figure 6B). The change in metabolite content showed significant changes in IAA and jasmonic acid in the rapid growth recovery variety B116, with a decrease in IAA level and an increase in jasmonic acid levels (Figure 5G). These results indicated that jasmonic acid and auxin jointly regulated the growth recovery of rice seedlings after combined stress from low temperatures and low light intensities, which was supported by changes in root length. The distribution of carbohydrates affects the formation of dry matter. In both varieties, the level of sucrose was significantly lowered (Figure 5G), with a higher reduction factor in B116 compared to B811, indicating that B116 could transfer sucrose more quickly, consistent with the change in root fresh weight. Similarly, the difference in gene expression confirmed this, with significant increases in sucrose phosphate synthase *SPS1* and sucrose synthase *SUS4* in both B116 and B811, with higher upregulation in B116 (Figure 6C). To further illustrate the changes in auxin and sucrose during growth recovery in the two varieties, we compared the metabolic levels of these two substances under different treatments. The results showed that the IAA and sucrose levels in B116 were significantly higher than those in B811. Even six days after normal light and temperature conditions were restored, the auxin and sucrose levels in B116 roots were still higher than those of B811, although they were significantly lower than those of the control (Figure 7). For the aforementioned genes in the metabolic process, we selected two auxin response proteins, one auxin response factor, one auxin amide synthase, and two sucrose metabolic genes for RT-qPCR validation. The experimental results showed that their expression levels were consistent with the trend in RNA-seq, further verifying our results (Figure 8).

## 3. Discussion

The quality of directly seeded rice at the seedling stage determines its growth and development in later stages and the final yield. The root system, as the main nutrient organ in the early growth process in rice, plays a crucial role in determining the growth of aboveground parts [15]. In this study, the combined stress from low temperatures and low light significantly decreased the number of main roots, total root length, total root surface area, and total root volume of rice compared to the control. The strong-growth-recovery variety B116 showed a rapid increase in total root length and the number of taproots after low-temperature and low-light stress, but the increase in root fresh weight was still lower than that observed in the control. On the other hand, the root system of B811 showed significantly low growth recovery [27,28]. Cold-sensitive rice root systems showed significantly higher decreases in surface area, volume, and weight after exposure to low temperature than low-temperature-tolerant varieties [29]. There were significant differences in the root growth recovery index and the ability of different materials to grow after stress. After combined stress from low temperature and low light, the number and length of roots increased first to ensure that there were enough nutrient absorption organs to compensate for the growth loss caused by stress, which was consistent with findings in previous studies [30]. Strong roots are important for rapid rice growth after low-temperature stress [31]. The rapid recovery period of the root system comprises the first three days after the stress is relieved, while the aboveground parts recover after 3–6 days [25]. This point has been mentioned in previous studies; after water stress, rice plants [32] entered a rapid-growth stage. Further studies are needed to understand how these characteristics can be utilized and how root thickness can be improved.

The results indicated that low-temperature stress during the bud and seedling stages had a significant inhibitory effect on the growth of rice roots due to the presence of ROS [7,33]. In this study, it was observed that the levels of H_2_O_2_ and MDA in the root systems of both varieties increased significantly under low-temperature and low-light stress, indicating a burst of ROS concentration in the root system. Upon the restoration of normal light and temperature conditions, as well as growth promotion, it was found that the ROS scavenging capacity of B116 was significantly faster than that of B811, which was attributed to higher ROS-metabolizing enzyme activities [34]. Based on the results depicting root growth and reactive oxygen metabolism, it can be inferred that the response of roots after exposure to combined stress from low temperatures and low light intensities was stronger than that of the aboveground parts. Following exposure to combined stress from low temperatures and low light intensities, the roots first entered a period of rapid growth recovery. Initially, the reactive oxygen compounds, represented by H_2_O_2_ and MDA, were removed, and then, the absorption area of roots expanded to absorb more nutrients for the overall growth recovery of plants [35].

Genes regulate various metabolic activities, and changes in gene expression levels in plants reflect alterations in growth metabolism to some extent [36]. Tolerant rice genotypes exhibit differences in biological regulation, signal transduction, photosynthesis, energy, and carbohydrate metabolism during different cold stress periods [37]. This study found that plant signaling and starch and sugar metabolism are associated with rapid growth recovery, and transcriptome analyses of the aboveground parts showed, similarly, the enrichment of plant signaling gene expression [25]. The metabolites of the two varieties were mainly enriched in metabolic pathways related to plant signal transduction and photosynthesis. By conducting correlation analysis with the metabolic pathways enriched by the transcriptome, we identified endogenous signal transduction and starch and sucrose metabolism as the key metabolic pathways. Further results showed that IAA/sucrose was the most important metabolite and was significantly associated with IAA signal transduction and sucrose-metabolism-related genes.

Maintaining appropriate levels of indole-3-acetic acid (IAA) is important for primary root elongation and lateral root development [38]. Auxin response proteins (AUX/IAA) can bind to auxin response factors (ARFs) and negatively regulate the expression levels of target genes [39,40,41]. The Aux/IAA signal response proteins IAA15 and IAA20 were downregulated, and IAA7 was upregulated after low-temperature and low-light stress. The ARF factors ARF11, ARF15, and ARF3 were significantly upregulated in both varieties. Interestingly, B116 consumed IAA at a faster rate after combined low-temperature and low-light stress (Figure 7). We speculate that during growth recovery, IAA depletion resulted in the significant downregulation of AUX/IAA proteins and significant upregulation of ARFs. This led to an upregulation of the expression of auxin amide synthase GH3.8 (Figure 8), which formed a more stable auxin conjugate that maintained IAA levels. It was revealed that the application of exogenous IAA increased the root surface area of rice by 26.4% after alkaline stress was relieved [42], and the expression levels of genes controlling IAA biosynthesis and transport were significantly increased. The amide complexes formed by IAA with amino acids are associated with a variety of abiotic stresses [43]. After low-temperature stress, starch degradation and sucrose metabolism enzyme activities are significantly activated to enhance energy metabolism [44]. In this study, B116 not only had a higher level of sucrose than B811 but also had a significantly faster level of sucrose downregulation after stress relief than B811. At the same time, the upregulation of sucrose synthase was significantly higher than that of B811, which is consistent with the findings that the rapid utilization of sucrose enhances low-temperature stress resistance in rice [45]. Therefore, we speculate that high levels of auxin and sucrose in roots may contribute to the rapid growth recovery of plants after low-temperature and low-light stress is relieved.

**Figure 9:**
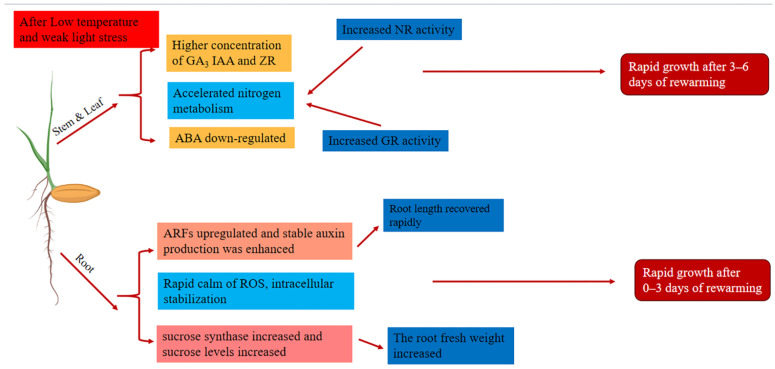
Growth recovery mechanism of rice seedlings during growth after low-temperature and low-light stress is relieved.

Based on the results of this study on roots and a previous study on stems and leaves, a model diagram of the rapid growth mechanism of rice seedlings after low-temperature low-light stress exposure was constructed (Figure 9). It should be noted that in this study, we focused on the damage caused by late spring cold to directly seeded rice. The growth recovery and related mechanism of the root system after low-temperature low-light stress was relieved, the main hazard factor in meteorological disasters was tracked, and no single stress factor was studied. Therefore, it is not clear which of the two stress factors, low temperature or low light, was dominant, although it is generally believed that low temperature is the main hazard factor [35]. Late spring cold is generally accompanied by rainfall and significant changes in humidity [25]. In this study, we made a good attempt to study root growth recovery in rice after exposure to cold stress in late spring.

## 4. Materials and Methods

### 4.1. Planting and Experimental Design

The study used B116 (R310/R974, F17), a conventional indica rice variety with 17 generations involving self-crossing of selected hybrid combinations by our research group, and B811 (Zhonghui 286), a conventional indica early rice variety, selected by China Rice Research Institute (CRI) (https://ricedata.cn/variety/index.htm, accessed on 25 October 2023). B116 is known for its rapid recovery after exposure to low-temperature and low-light stress, while B811 is a slow-growth-recovery variety. Both experimental varieties are homozygous and stable inbred lines, and both are maintained by our laboratory and were planted in the experimental field.

The experiment took place from March to September 2022 in Nanchang City (115.8 E, 28.7 N), Jiangxi Province, China. Prior to sowing, the seeds were selected and soaked in local production model solutions to induce germination until they were exposed to stress. Then, the seeds were evenly spread in plastic barrels each with a diameter of 33 cm at the top, 23.5 cm at the bottom, and 25 cm in height. The soil used in the experiment was shallow cultivation soil from a rice field, which was collected, stacked in the base yard for natural drying, crushed using an FT-1000A soil crusher (Shandong Anbo Instrument Co., Ltd. Shandong, China), and passed through a 100-mesh sieve. Each barrel was filled with 25 kg of air-dried soil, and 1 g compound fertilizer (N-P-K = 15%-15%-15%) was applied.

The basic physical and chemical properties of the tested soil were as follows: the soil pH value was 5.3, the organic matter content was 31.7 g/kg, and the available nitrogen, phosphorus, and potassium contents were 108 mg/kg, 18.2 mg/kg, and 105.8 mg/kg, respectively. The experiment had a 12 h cycle (6:00–18:00). The control group (CK) had a light intensity of 533 µ mol m^−2^s^−1^, with daytime (6:00–18:00) and nighttime (18:00–6:00) temperatures of 27 °C and 25 °C, respectively and a relative humidity of 75%.

Late spring cold was simulated based on spring low-temperature events and late-spring-cold standards in southern China in the past 40 years [46], and the effect of combined stress from low-temperature and low-light conditions was determined. The low-temperature treatment was conducted at a daily average temperature of 12 °C, with daytime (6:00–18:00) and nighttime (18:00–6:00) temperatures of 14 °C and 10 °C, respectively. The light intensity was 266 µmol m^−2^s^−1^, 50% of that in the control, and the relative humidity was 75%, consistent with the control. After the rice seedlings grew to two leaves and one heart at normal temperature, they were subjected to an average low-temperature treatment of 12 °C for 4 days, and then, the temperature was rapidly changed to a normal growth temperature of 26 °C and a daily light intensity of 533 µmol m^−2^s^−1^. During the normal temperature and light intensity stage, the root growth status of the rice seedlings was investigated, and roots were sampled.

### 4.2. Investigation of Root Growth Status

On the 0th, 1st, 3rd, and 6th days after returning to normal temperature and light intensity conditions, the status of root growth was examined. Five plants with consistent and uniform growth were selected for each treatment, and their roots were cleaned and placed flat on a scanner board. The roots were scanned and imaged using an EPSON Expression 12000XL scanner (Epson (China) Limited, Shanghai, China). The root analysis system WinRHIZO Pro (2019a, Quebec, Canada) [47] was used to analyze and assess the root growth status, including total root length, total root surface area, and total root volume.

### 4.3. Root Reactive Oxygen Metabolism

On the 0th, 1st, 3rd, and 6th days after returning to normal temperature and light intensity conditions, samples were collected from the roots of rice. Roots with uniform growth were selected, and the soil was washed off. The roots were rinsed two to three times with PBS buffer and stored at −80 °C for testing. Each treatment was repeated three times. Approximately 0.1 g of root samples was taken and ground, and 10 mL of extract was added. After centrifugation, the supernatant was collected and measured. SOD activity was measured using the NBT assay kit (Suzhou Keming Biotechnology Co., Ltd.Jiangsu, China. SOD-2-Y). POD activity was determined using the catalysis of hydrogen peroxide by catalase using a kit (Nanjing Jiancheng Bioengineering Institute, Nanjing, China. A084-3-1). CAT activity was determined using an ammonium molybdate assay kit (Nanjing Jiancheng Bioengineering Institute, Nanjing, China. A007-1-1). H_2_O_2_ was measured using a kit based on the principle that it forms a complex with molybdate (Nanjing Jiancheng Bioengineering Institute, Nanjing, China. A064-1-1). MDA content was determined using a TBA kit (Nanjing Jiancheng Bioengineering Institute, Nanjing, China. A003-1). Soluble sugar content was determined using an anthrone colorimetric kit (Nanjing Jiancheng Bioengineering Institute, Nanjing, China. A145-1-1).

### 4.4. RNA-seq Assay Analysis

Total RNA was extracted using a TRIzol reagent kit (Invitrogen, Carlsbad, CA, USA) following the manufacturer’s protocol. RNA quality was assessed on an Agilent 2100 Bioanalyzer (Agilent Technologies, Palo Alto, CA, USA) and checked using RNase-free agarose gel electrophoresis. After total RNA was extracted, eukaryotic mRNA was enriched with oligo(dT) beads. Then, the enriched mRNA was fragmented into short fragments using a fragmentation buffer and reverse-transcribed into cDNA by using the NEB Next Ultra RNA Library Prep Kit for Illumina (NEB #7530, New England Biolabs, Ipswich, MA, USA). The purified double-stranded cDNA fragments were end-repaired, a base was added, and the fragments were ligated to Illumina sequencing adapters. The ligation reaction was purified with AMPure XP Beads (1.0X). In addition, polymerase chain reaction (PCR) amplification was performed. The resulting cDNA library was sequenced using Illumina NovaSeq 6000, manufactured by Gene Denovo Biotechnology Co. (Guangzhou, China). RNA differential expression analysis was conducted using DESeq2 [48] software between two different groups (and by using edge-R between two samples). Genes/transcripts with a false discovery rate (FDR) below 0.05 and an absolute fold change ≥ 2 were considered differentially expressed genes/transcripts.

Pathway-based analysis was used to further elucidate the biological functions of genes. The KEGG database was utilized for pathway enrichment analysis, and it identified significantly enriched metabolic pathways or signal transduction pathways in DEGs compared with the whole genome background. The calculated *p*-value was subjected to FDR correction, with an FDR ≤ 0.05 considered as a threshold for significantly enriched pathways in DEGs.

### 4.5. Extraction of Metabolites

After slowly thawing the sample at 4 °C, an appropriate amount was taken and a precooled solution of methanol, acetonitrile, and water solution (2:2:1, *v*/*v*) was added. The sample was then vortexed and ultrasonicated at −20 °C for 10 min and centrifuged at 14,000× *g* at 4 °C for 20 min. The supernatant was vacuum-dried, and 100% acetonitrile aqueous solution (acetonitrile:water = 1:1, *v*/*v*) was added for mass spectrometry analysis. The sample was redissolved, vortexed at 14,000× *g*, and centrifuged at 4 °C for 15 min, and the supernatant was used for injection analysis. The quality control sample (QC) was a mixture of equal volumes of the samples to be tested, which was used to determine the state of the instrument before injection, balance the chromatography‒mass spectrometry system, and evaluate the stability of the system during the entire experimental process.

### 4.6. Identification and Differential Analysis of Metabolites

Compounds were separated using an Agilent 1290 infinite LC HILIC column at a temperature of 25 °C, with a flow rate of 0.5 mL/min, and an injection volume of 2 µL. The mobile phase consisted of A: water + 25 mM ammonium acetate + 25 mm ammonia water and B: acetonitrile. Samples were stored in a 4 °C autosampler throughout the analysis process. After HILIC chromatographic separation, an AB triple TOF 6600 mass spectrometer was used to collect primary and secondary spectra of the samples. The original data were converted to an MzMl format using ProteoWizard, and then, the XCMS (https://xcmsonline.scripps.edu) program was used for peak alignment, retention time correction, and peak area extraction. The integrity of the data extracted by XCMS was checked, and metabolites with more than 50% missing values in the group were removed from subsequent analysis. Null KNN values were filled, extreme values were deleted, and the total peak area of the data was normalized to ensure parallelism between samples and metabolites. Material annotation was performed based on Mass Bank, Metlin, Mona, and other public databases, as well as a secondary mass spectrometry database custom-built by Gene Denovo Biotechnology Co., Ltd. (Guangzhou, China).

The VIP (variable importance for the projection) value was used to determine the importance of the characteristic peak in explaining the importance of the X dataset and its association with the Y dataset. The VIP value from the multivariate statistical analysis OPLS-DA [49] and the *p*-value from the univariate statistical analysis t test were combined to screen for differential metabolites between different comparison groups [50]. The threshold for difference was VIP ≥ 1 and *t*-test with *p* < 0.05 in the OPLS-DA model.

### 4.7. RT-qPCR

To validate the accuracy of the transcriptional sequencing results, real-time fluorescent quantitative PCR was used to identify differentially expressed genes. Primers were designed using Snapgene software (v4.1.9) (Table S5). The Chq general SYBR^®^QPCR main mixture q711 (Vazyme Biotechnology Co., Nanjing, China) was used. The RNA used for real-time fluorescent quantitative RT-qPCR was the same as that used to construct the cDNA library, and the expressions of the related genes were normalized to the actin transcription level via the 2^−∆∆ ct^ method.

### 4.8. Data Analysis and Chart Construction

Data analysis was conducted using Excel and SPSS 19.0. The significance of differences was analyzed using Duncan’s analysis at a significance level of *p* < 0.05. Graphics were created using GraphPad Prism. 8.0.

## 5. Conclusions

Combined stress from low-temperature and low-light conditions significantly inhibited root growth, with the most significant decrease being in root length. Once normal light and temperature conditions were restored, genotype B116, known for strong growth recovery, exhibited rapid root growth recovery, with root length and main root number values recovering first. Peak root growth occurred within the first three days after normal light and temperature conditions were restored, while recovery in the aboveground parts was delayed by three days. The auxin signal transduction pathway and starch and sucrose metabolism pathways were closely related to growth recovery. According to the results of RT-qPCR, it was suggested that the auxin response factor and auxin amide synthase jointly regulated growth and recovery in roots. In conclusion, the rapid growth recovery of rice seedlings after combined stress from low-temperature and low-light conditions was associated with high root auxin and sucrose content, the rapid removal of reactive oxygen species, the rapid consumption of auxin, and the rapid metabolism of sucrose. Additionally, the rapid synthesis of sucrose was key to the rapid growth and recovery of roots. These results provide a theoretical basis for breeding rice with the ability to rapidly recover from growth inhibition after exposure to combined low-temperature and low-light stress, as well as for optimizing cultivation regimes.

## Data Availability

The transcriptome data generated in this study have been deposited in the NCBI SRA database under BioProject PRJNA1007370. The generated dataset can be queried through links: SRP456403–SRA–NCBI (nih.gov). This project includes a total of 12 data, with accession in order: SRX21452739, SRX21452738, SRX21452737, SRX21452736, SRX21452735, SRX21452734, SRX21452733, SRX21452732, SRX21452731, SRX21452730, SRX21452729, SRX21452728.

## Supplementary Materials

The following supporting information can be downloaded at: https://www.mdpi.com/article/10.3390/ijms242417359/s1.
